# College Notes

**Published:** 1894-12

**Authors:** 


### COLLEGE NOTES.

Dr. Carter, of the Medical Department of the University of Pennsylvania, has succeeded to the Chair of Physiology in the Veterinary Department, vice Dr. Leo Breisacher, whose resignation was accepted, to enable him to take up practice in New York City.

The New York College of Veterinary Surgeons finds their new home enables them to add very much to the scope and effectiveness of their course of instruction.

The closer affiliation of the Montreal Veterinary School as a Department now of McGill University is proving a great step of advancement, and adding to the better success and strengthening of the course of instruction given at that school. It also gives a support and protection to that school that it did not possess before its union with McGill.

At the Veterinary Department of the University of Pennsylvania, the '94 class numbers between thirty and forty, which is quite up to the number of the class of '93, which is still another evidence that in the general shrinkage of students, at the various colleges of our country, those which have the broadest and most extensive curriculum have not suffered in proportion to those which have very short terms as inducements for new recruits to their classes.

California, in January next, will open a new Veterinary School, which will be in some way associated with the California State University, though the funds necessary for its establishment have been raised by private subscription. It is announced that no less than fifty students will enter at the opening of the session. This should be a good field for a Veterinary School, but it should be on a very high standard, and it is hoped that they will not start with a curriculum of less than three years of six months each.

It is interesting at this time to know that the proposed Veterinary Department of the Rocky Mountain University, at Denver, Colorado, which was in contemplation in the fall of 1890, was to start out with a three years' course. The third year course of lectures in the school were to be given free of cost.

Graduates of the Veterinary Department of the Ohio State University now number fifteen, four of whom hold positions as Instructors in Veterinary Schools, or are connected with Agricultural Experiment Stations.

Truly the signs of the times in Veterinary Education bear continued evidence of the result of the advancement the last two years. We note, with great pleasure, in the columns of our contemporary, *The Review*, the agitation for a higher curriculum in the Toronto School and its being placed under the government control of Ontario. This agitation arises in the best plac it could possibly arise, that is to say, among the Alumni of that institution.

A change was made at the beginning of the year in the Ohio Veterinary College at Cincinnati, in the resignation of Dr. N. B. Jones, Dean, and the appointment of his successor, Thomas King, a graduate of the Ontario Veterinary College, also chosen to fill the Chair of Theory and Practice. Dr. Jones showed great interest in the movement to advance the curriculum of Veterinary Colleges in North America, and we hope that the good work which he planned for their school will be more than carried out by his successor.

During the present collegiate term at Toronto Veterinary College, no special changes have taken place, only a more determined effort to make more thorough the course, and greater use of the increased facilities which they obtained some two years ago. They report a much better educated class of students for entrance this year.

Dr. W. H. Ridge, Lecturer on Obstetrics at the Veterinary Department of the University of Pennsylvania, has resigned. Dr. M. E. Conard, of West Grove, Pa., has been appointed in his place.

The preliminary announcement of the California Veterinary College has been presented to the public. They expect to commence their initial work on the 7th of January, 1895, after which the sessions will regularly convene in October. Associated with the enterprise are Drs. Bowhill, Buzard, Egan, Nief and Skaife, well known Veterinarians of the Pacific Coast. The adjunct faculty has not yet been announced. The degree of Doctor of Veterinary Science will be conferred. A curriculum of three years of six months each will be required as a course.

The National Veterinary College made, during the present collegiate year, several changes in the faculty. Dr. John Lockwood succeeded the late Dr. C. B. Michener as Profesfor of Principles and Practice of Medicine and Clinical Medicine. Dr. E. R.

Hodge was appointed to the Chair of Chemistry, vice Prof. E. A. de Schweinitz. A course in Medical Jurisprudence was added to the course of lectures, and are being given by Hon. Edwin Willits. A course in Practical Farriery has been added also this year. The entrance requirements call for a common school education, which gives great latitude in the determination of what this shall consist of. The senior, or graduating class, numbers fifteen, while the new class, or Juniors, number eight.

There have been no special changes at the Veterinary Department of the Iowa Agricultural College the present year. Their Senior class contains some thirteen students, while the Junior class contains nine. The number in the Freshman class we have not been able to obtain. We note that the faculty, as announced in the catalogue of '94, designates but three veterinarians on the teaching staff.

On Wednesday evening, December 12th, the Philadelphia Veterinary Society, in disposing of its regular order of business, was entertained by Prof. S. J. J. Harger, in a description of the conformation of the head and cranial cavity as an indication of intelligence, particularly from an anatomical point of view, and was listened to with much pleasure by all those in attendance; after which W. W. McGuire exhibited his famous pony, "Mazeppa," and demonstrated before the audience the wonderful degree of intelligence which this animal seems to possess; and to those who were present it was a revelation, mystifying as well as interesting, to see the animal, by the head and feet, tell the time by watches, count up the age of any in attendance, add up columns of figures, subtract one number from another, as well as the announcement of figures, under circumstances that would seem almost beyond belief.

It will be interesting to our readers to learn that the twelfth year of instruction at the Veterinary Department of Harvard University has in her various classes sixty-two students this year, which is an increase of about forty within three years, and this, too, under one of the highest entrance examinations of any veterinary school. A curriculum of three years, of eight months each, and a high standard for graduation, with the total fees $150 per session. It should be said in this connection, though, that this covers the matriculation and diploma fee, or, in other words, covers the cost of the entire instruction given. Her teaching staff now numbers twenty-one instructors, nine of whom are veterinary surgeons.

The subjects taught embrace anatomy, divided into an elementary and advanced course, covering two years; physiology; chemistry, general and medical, covering two years; botany; materia medica; comparative pathology; the inspection of meat, and special instruction in making autopsies; surgery; surgical pathology; operative veterinary surgery, with special instruction in the application and use of bandages and apparatus; ophthalmology of the domesticated animals, supplemented by a valuable, specially arranged course of clinical instruction at the eye and ear infirmary; a thorough course on parasites and parasitic diseases; the theory and practice of veterinary medicine, including the diseases of horses, cattle, sheep and dogs, each given by a special instructor; obstetrics, a special course; warranty and evidence, including a general discussion of the rules of evidence, with practical suggestions on expert testimony and the conduct of witnesses in court, with practical talks upon the law of sale and warrantee; practical work in the various laboratories, with six clinical exercises a week, each given by a different instructor, upon cases in the veterinary hospital.

This would seem to be an ideal course, and certainly must be a very great factor in advancing the standard of veterinarians at home and abroad, in the very near future.

Mr. J. H. Monrad, editor of the *National Dairyman*, has been added to the list of instructors on practical milk inspection, at the Kansas City Veterinary College. The school this year has fifteen in the senior class, and twelve in the junior class. Five of the latter have matriculated for the three years' course. All matriculants were required to conform to the first four rules for admission now exacted at the Veterinary Department of Harvard.

The American Veterinary College has enrolled this year 101 students. One taking a post-graduate course, twenty-seven in the third year's class, thirty-four in the second year, and thirty-nine in the first year. There is an increase of twenty-five per cent. in the first year class of '94 over that of '93, which is certainly a strong recommendation for an advanced curriculum, and must be very gratifying to the managers of that well-known school.

The friends of the New York College of Veterinary Surgeons, and the profession in general, will be very glad to learn of the added strength to the teaching staff and the curriculum of this school. As announced, after January 1st, all matriculants will be compelled to enter for a three years' course. There have been added to the teaching staff Dr. R. W. Hickman, one of the Inspectors of the Bureau of Animal Industry, and Dr. Lellman, graduate of Berlin, upon whom was recently conferred the doctorate degree by the University of Giessen. In their new location and more commodious quarters they have had a very great increase in their hospital work. Their well-equipped clinic and operating room affords them greater opportunities for more thorough work. Their new lecture room, arranged to seat 250 students, is so constructed that lantern work may be used for the better instruction of the students in displaying upon the screen microscopical and other work. Chemical, histological, pharmaceutical and bacteriological laboratories have been provided with excellent apparatus for instruction. At present, the Health Department of the City of New York is using their laboratory for the preparation of the antitoxine of diphtheria. Septic and anti-septic surgery, as applied to veterinary practice, is receiving a special series of lectures; while meat and milk inspection and veterinary hygiene, including the methods of diagnosis with tuberculin and mallein, are also a part of the present course. All of which must surely tend to the greater advancement of American veterinary science, and places her schools on a high plane of work.

